# Development of a chemical-free floatation technology for the purification of vein graphite and characterization of the products

**DOI:** 10.1038/s41598-021-02101-9

**Published:** 2021-11-22

**Authors:** Gamaralalage R. A. Kumara, Herath Mudiyanselage G. T. A. Pitawala, Buddika Karunarathne, Mantilaka Mudiyanselage M. G. P. G. Mantilaka, Rajapakse Mudiyanselage G. Rajapakse, Hsin-Hui Huang, K. Kanishka H. De Silva, Masamichi Yoshimura

**Affiliations:** 1 National Institute of Fundamental Studies, Hantana Road, Kandy, 20000 Sri Lanka; 2 Postgraduate Institute of Science, University of Peradeniya, Peradeniya, 20400 Sri Lanka; 3 Department of Chemistry, University of Peradeniya, Peradeniya, 20400 Sri Lanka; 4 Graduate School of Engineering, Toyota Technological Institute, 2-12-1 Hisakata, Tempaku, Nagoya, 468-8511 Japan; 5 Department of Geology, University of Peradeniya, Peradeniya, 20400 Sri Lanka

**Keywords:** Chemistry, Materials science

## Abstract

A novel and simple flotation technique has been developed to prepare high-purity graphite from impure graphite. In this method, a suspension of pristine powdered graphite (PG) is dispersed and stirred in water without adding froth formers or supportive chemicals. This makes fine particles of graphite move upwards and float on water. X-ray diffraction (XRD) analysis reveals that the floated graphite (FG) has a lower c-axis parameter, indicating the removal of interlayer impurities. A notable increase in the intensity ratio of the D band to G band in the Raman spectra indicates that the FG has more edge defects due to their smaller crystallite sizes. Transmission electron microscopic (TEM) analysis shows the number of layers in FG has been reduced to 16 from 68 in PG. The absence of C=O vibration of Fourier Transformed Infrared (FT-IR) spectroscopy in treated and untreated samples suggests that their layers are not significantly oxidized. However, X-ray photoelectron spectroscopic (XPS) analysis shows the presence of C–O–C ether functionalities, possibly on edge planes. Further, the product has higher purity with increased carbon content. Therefore, the technique is helpful for the value enhancement of graphite, the reduction of the chemical cost of the conventional techniques, environmental friendliness, and improvement of its applications.

## Introduction

Graphite is one of the important naturally occurring minerals characterized by its inherent specific properties such as low hardness, metallic lustre, high lubricity, refractoriness, high heat and electrical conductivities, and ability to withstand to high temperatures. These properties of graphite make it an attractive material for many technological applications^1,2^. Naturally occurring graphite has a wide range of purities depending on its geological occurrence^3–5^.

Demand for high purity graphite has been progressively increasing in the recent past due to its versatile traditional applications and numerous novel applications that researchers foresee, in the near future^4,6,7^. Naturally occurring graphite is classified into three forms: (i) flake graphite, (ii) vein graphite and (iii) amorphous graphite^8^. Flake graphite is found in regionally metamorphosed sedimentary rocks and has a distinctly flaky morphology, and is typically found as flat and plate-like masses^9^. They are very common in many parts of the world. However, vein graphite is found only in Sri Lanka, India, Madagascar, the USA, Canada and the UK^10–12^.

Although the carbon content of vein graphite varieties is considered to be high up to about 90–99.8%, their natural purity is often in the lower-end of this range due to the presence of gangue minerals which are naturally associated with the graphite veins^5,13^. Furthermore, the purity of natural vein graphite varies from place to place in the same deposit. Impurities are present as mineral inclusions, or they are intercalated between graphite layers. The major impurities of vein graphite are Fe, Ca, Mg, Si, Al and Na with minor to trace concentrations of transition metals such as Cu, Ni, Co and Zn^14^. The presence of impurities inevitably decreases the quality of even such highly pure vein graphite. Thus the development of simple, low-cost, scalable and industrially-viable purification techniques is still required for better industrial utilization of natural graphite.

Several methods have been introduced to enhance the purity of graphite powders but the majority of them are based on environmentally unfriendly acid or alkali treatments^15^. In contrast, other methods involve expansion processes and physical treatments^16^. Among these techniques, floatation is an industrially adaptable, simple, cost-effective and selective mineral-processing technique^17^. The raw material concentration by floatation generally utilizes the surface physicochemical properties of water-repellent (hydrophobic) particles to enable them to float with air bubbles in order to form a froth^18^. Together with the froth the fine particles move upwards to float on the water surface. In the absence of considerable amounts of functional groups such as ether, hydroxyl and carboxyl formed by the ariel oxidation of valence-unsatisfied surface carbon atoms of graphite crystals, naturally occurring graphite forms with high carbon percentages are generally hydrophobic. The hydrophobicity of graphite particles, together with some added specific chemical reagents to help improve froth formation, is used in the purification of mined graphite^19^.

Although graphite has specific gravity around 1.9–2.3, the hydrophobic nature of fine graphite particles aids the floatation process when water is used as the floating medium. The contact angle in the air–water–mineral system measures the hydrophobicity of a mineral surface and graphite is characterized by relatively large contact angles that depend, to some extent, on the pH of the water used and also on the characteristics of the surface preparation^17^. Graphite floatation is not new and has been already studied by several researchers^19–21^. However, most of these studies have focused on the chemical composition of the floated graphite, particularly, on the total carbon content and trace elements. But the structural and morphological characteristics of floated graphite, which are key properties for industrial applications, have not been evaluated. In graphite floatation, oils such as kerosene^22^ and diesel^23^ combined with froth formers are used to enhance froth formation. The use of such chemicals undoubtedly complicates the purification process since all these chemicals that may have bound or adsorbed on graphite crystallites have to be removed at the final stage. Usage of chemicals has increased environmental problems due to the generation of wastes, high production costs and consequent reduction of profits. This process demands not only chemicals but also intensive labour, making it industrially less attractive. Also, most of the froth formers used in the floatation of graphite are environmentally unfriendly chemicals. Hence a method that does not require any chemical to float graphite on the water will be more industrially-viable and adaptable.

In this manuscript, we reveal an effective chemical-free, simple floatation technique developed to obtain high-purity and well-crystalline graphite products by floating fine particles of graphite on water. The pristine powdered graphite (PG) and floated graphite (FG) are characterized using several independent material characterization techniques. The method’s success is explained in terms of improved physical and chemical properties of FG. This method is scalable, simple and of low-cost, making it industrially viable, adaptable and environmental-friendly. The developed method has enhanced the purity of graphite from 91.9 to 98% of carbon content which makes a 4–12 times value addition to raw-graphite. Therefore, the developed chemical-free floatation technique is beneficial for the value enhancement of graphite, reduction of chemical cost involved in the conventional froth floatation techniques and environmental friendliness.

## Results and discussion

Out of 5.0 g of PG taken for floating in water 4.5 g of FG was recovered. Thus, the process of converting PG to FG has 90% efficiency. The reaming 10% is due to the removed inclusion mineral impurities together with some larger particles that are too heavy to float in water. Since we have limited the ball-milling time to a short 20 min. there is a very small amount of larger particles remaining in the PG samples. Out of the 10% the majority is the removed inclusion mineral impurities.

**Figure 1:**
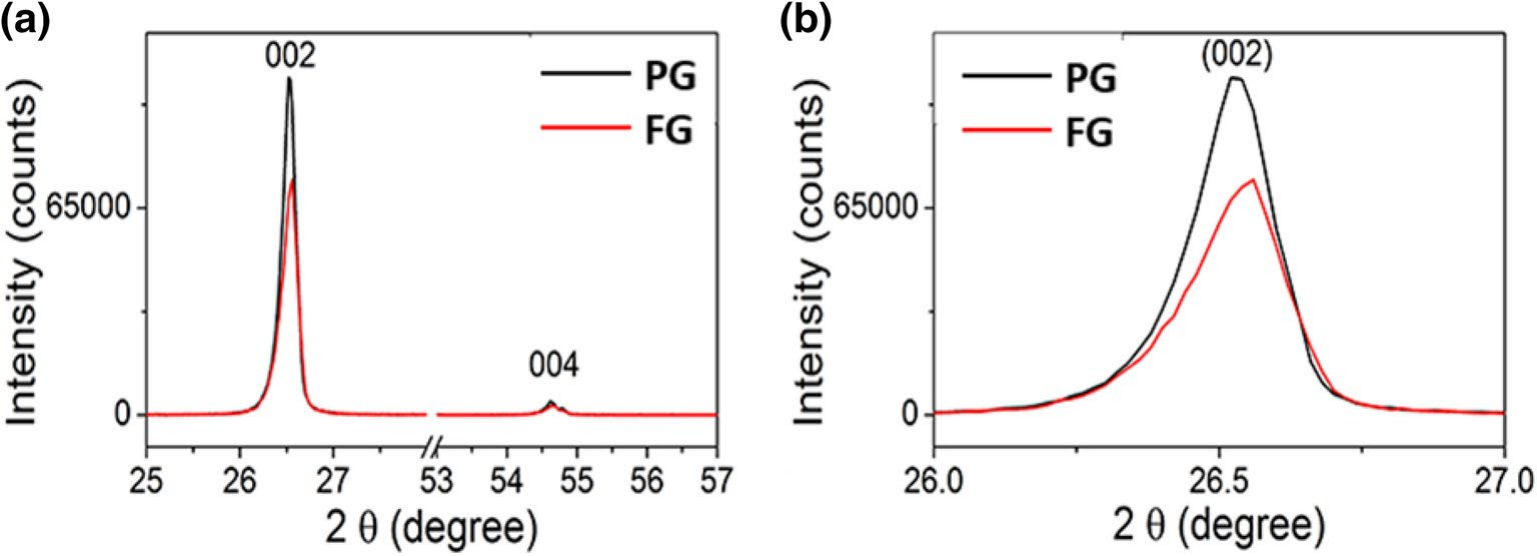
(**a**) XRD patterns of the PG and FG samples. (**b**) The enlarged (002) reflections used for the calculation of crystallite size.

Figure 1 shows the XRD patterns of the PG and FG samples. The intense (002) diffraction peak appearing at 2θ = 26.52° of PG has been shifted to 26.56° in FG indicating that the c-axis lattice parameter of the FG to be smaller (0.3353 nm) than that of the PG (0.3358 nm). Moreover, the FG’s full width at half-maximum (FWHM) of the (002) XRD peak is significantly larger than that of PG. Application of the Scherer equation to the FWHM of the (002) peak gives the crystallite size of the FG and PG to be approximately 86 nm and 101 nm, respectively. However, the intrinsic instrumental broadening should also be considered when elucidating exact values. A slight decrease in interlayer spacing suggests the removal of interlayer species that are present in low quantities in pristine graphite samples. As such, XRD provides indirect evidence to show that the floating technique can aid the enrichment of carbon percentage in graphite samples. It has been proven by the quantitative elemental analysis of the two samples (vide infra). The XRD analysis of flake graphite (99% Purity, Alfa Aesar) done by Abdolhosseinzadeh et al. shows a very similar pattern where the peak positions of flake graphite have been shifted to higher 2θ value of 26.7° giving a d-value of 0.3340 nm. This is a further evidence to show the effect of purity on lattice parameters of graphite crystallites. Although absolute values of intensities (I_s_) of XRD depend on the amount of diffracting atoms present in the sample, which in turn depends on the amount of material used to get the diffractograms, the normalized intensities obtained by dividing I_(002)_ by I_(004)_ are meaningful and independent of the amount of materials used. As revealed by XRD data, the floating of ball-milled graphite has increased this intensity ratio indicating that floating of ball-milled graphite has resulted in increased crystallinity of the sample. The decreased crystallite size and the decreased interlayer distance of the FG collectively indicate the shrinking of particles due to floating, which may be due to the removal of impurities in the pristine graphite when it is milled and floated in the water.

**Figure 2:**
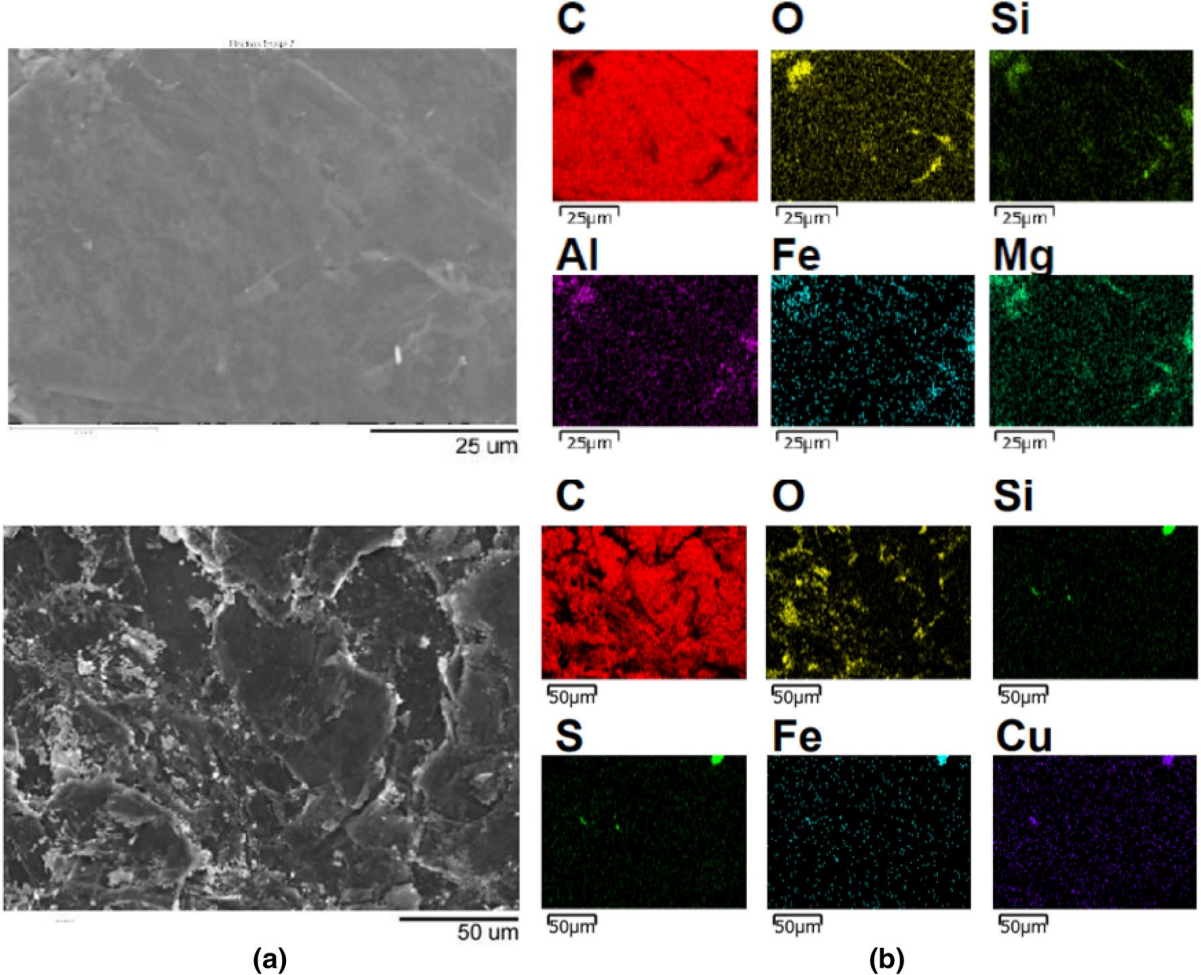
The (**a**) SEM images of pristine graphite samples collected from different locations and (**b**) EDX images showing elemental distributions of graphite in the two samples.

Figure 2 shows the SEM images and atomic distribution as determined by the EDX images of two pristine graphite samples obtained from different location without powdering. It is clear that the impurities of the graphite samples, determined by EDX, are different in terms of their composition and distribution. The C EDX shows full coverage since C is the major element present in graphite. The O distribution is shown in yellow patches and dots. This indicates that O is present in mineral inclusions and not attached to C to form graphite oxide. Further, the distribution of other elements indicates the presence of several mineral inclusions. Therefore, it is very likely that O is present as Al-bearing silicates which may also contain Mg and Fe also. Additionally, Fe and Cu can exist as their oxides, sulphites or mixed oxide/sulphides. There is a possibility for Cu to present as native Cu also.

The compositions of FG and PG samples determined by the SEM-EDX and IPC-MS are given in Tables 1 and 2, respectively. As can be seen from Table 1, the C atomic percentage has been increased by 7.86% when PG is converted to FG showing a significant enrichment of the carbon content. At the same time, the O and Si atomic percentages have been decreased by 5.76% and 2.16%, respectively. However, the EDX results show higher atomic percentages of S, Mg, Fe, Al and Cu in FG than those in PG. In order to explain the increase in the atomic percentages of some elements, it is important to consider the nature of the impurity elements present in the graphite samples. The impurities in vein graphite can occur either as mineral inclusions such as quartz, pyroxene, pyrrhotite, pyrite, chalcopyrite, sphalerite, marcasite, chlorite, calcite, siderite, dolomite, copper or as elements incorporated into the crystalline lattice^10,14,24^ Depletion of elements in low-carbon graphite indicates that most of the impurities exist as mineral inclusions. Some of these mineral inclusions are removed during the flotation as can be seen by the decreased atomic percentages of Si and O. This shows that some silicate minerals included in graphite have been removed by the flotation. However, there are some minerals still remaining in the FG indicating that it contains fine inclusion of some minerals, as shown in the EDS images given in Fig. 2. Therefore, silicate minerals such as quartz (SiO_2_), pyroxene group minerals (Mg,Fe)SiO_3_ or (Ca_x_Mg_y_Fe_z_)(Mg_y1_Fe_z1_)Si_2_O_6_) and chalcopyrite (CuFeS_2_), as well as native element minerals such as copper (Cu) and iron (Fe) can be suggested from EDS elemental distribution of original graphite sample.

**Table 1:** Elemental analysis of graphite samples by EDS.

Elements	C	O	Si	S	Mg	Fe	Al	Cu
Sample FG mass%	96.54	2.66	0.23	0.18	b.d	0.26	0.12	b.d
Sample FG atomic %	97.67	2.02	0.10	0.09	b.d	0.06	0.06	b.d
Sample PG mass%	84.69	9.77	4.99	b.d	0.18	0.22	n.d	0.19
Sample PG atomic %	89.81	7.78	2.26	b.d	0.06	0.05	n.d	0.04

*b.d* below the detection limits, *n.d* not detected.

**Table 2:** Compositions of the graphite samples as determined by ICP-MS analysis.

Sample	Purity	Carbon content together with some minor elemental impurities as mass%						
		Al	K	Fe	Ca	Mg	C_total_	
PG	High	< 0.01	< 0.01	0.03	0.05	0.01	95.9	
FG		< 0.01	< 0.01	0.09	0.01	0.01	98.0	
PG	Moderate	0.04	< 0.01	0.11	< 0.01	0.06	92.9	
FG		0.04	< 0.01	0.12	< 0.01	0.06	97.3	
PG	Low	0.14	< 0.01	0.21	0.09	0.14	91.9	
FG		0.09	0.01	0.14	0.03	0.09	98.0	

Sample	Purity	Trace elements in ppm (abundance is too low to be given as mass %)						
		Ba	Bi	Cr	Cu	Mn	Mo	Pb
PG	High	7.3	1.7	6	24.6	6	0.2	1
FG		11.7	1.6	4	19.7	7	0.1	1
PG	Moderate	7.7	1.7	1	38.5	7	0.3	1.3
FG		8.1	1.6	< 1	38.1	7	< 0.1	1.5
PG	Low	6.2	0.4	14	19	31	0.6	1.3
FG		11.8	0.2	8	10.1	20	0.3	0.9

Further, some silicate minerals with copper and iron are also present in raw samples. These minerals may have formed due to the alteration processes of the hydrothermal fluids. However, the carbon percentage has increased to 97% from 86% in the original mineral, due to the removal of silicate minerals during the flotation process. However, the fine inclusion such as iron-bearing and silicate minerals have not been fully removed, and they have floated with graphite (Table 2) as some of them occur as nanometer-scale minerals (see Fig. 2b). Since the EDX calculates the mass percentages by considering available elements it is possible to show higher values when the number of different elements is less. This may be the reason for the increased atomic percentages of Fe, Al and S shown in FG samples. The enrichment of Ba in floated graphite indicates that the element is incorporated into the crystalline lattice. However, the final FG products in all products contain over 97.3% of carbon content (Table 2). Therefore final FC products are suitable for applications including nuclear reactors, furnaces, advanced materials, specific niche applications, expandable graphite products, composites, and electronic applications. The final products can be marketed for USD 4000–6000 per tonne as per the graphite market in 2019^4^ whereas the PG with 91–95.9% carbon content has market value of USD 500–1100 per tonne. Therefore, the simple floatation technique reported in this study gives a considerable high value addition of 4–12 times. This can be further increased by making even smaller PG particles and utilizing processes such as magnetic separation to remove iron in final FG products.

**Figure 3:**
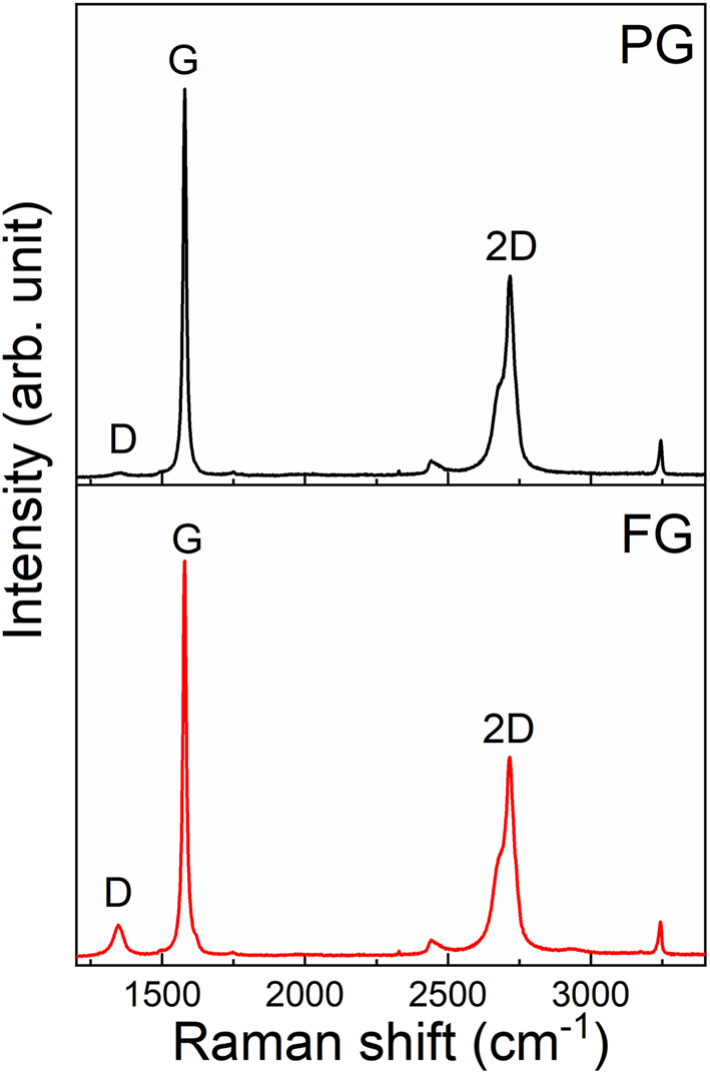
Raman spectra of (**a**) powdered graphite (PG) and (**b**) floated graphite (FG) samples. The D peak appears in FG sample due to the edge effects.

The Raman spectra given in Fig. 3 show three prominent bands centred at 1347 cm^−1^, 1579 cm^−1^, and 2690 cm^−1^ which correspond to the D, G, and 2D bands of both PG and FG samples, respectively. Interestingly, the band positions have not been changed due to crystallite size reduction. However, the intensity of the D band of FG is higher than that of PG and the intensity ratio of the D band and G band (I_D_/I_G_), of FG and PG are 0.10 and 0.03, respectively. The increased intensity of the D band is due to increased defects in the sample. The floating has reduced the crystallite size by removing some mineral inclusions and during this process more edge defects have been formed.

**Figure 4:**
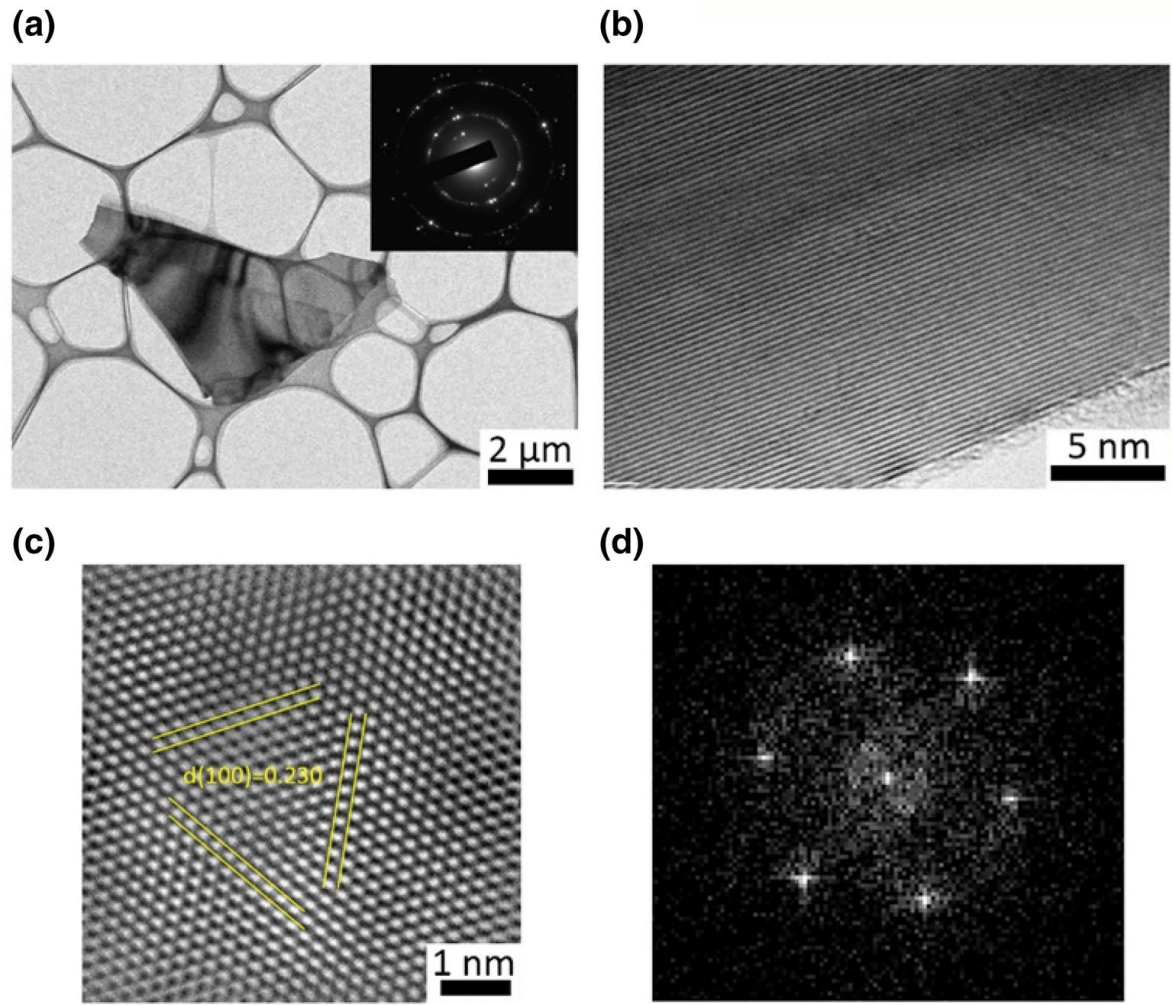
(**a**) Low-magnification bright-field TEM image of the PG specimen with an inset of corresponding [0001] electron diffraction pattern. (**b**) TEM image of the folded edges for the PG specimen. (**c**) Fourier filtered high resolution TEM image and (**d**) the corresponding FFT image.

**Figure 5:**
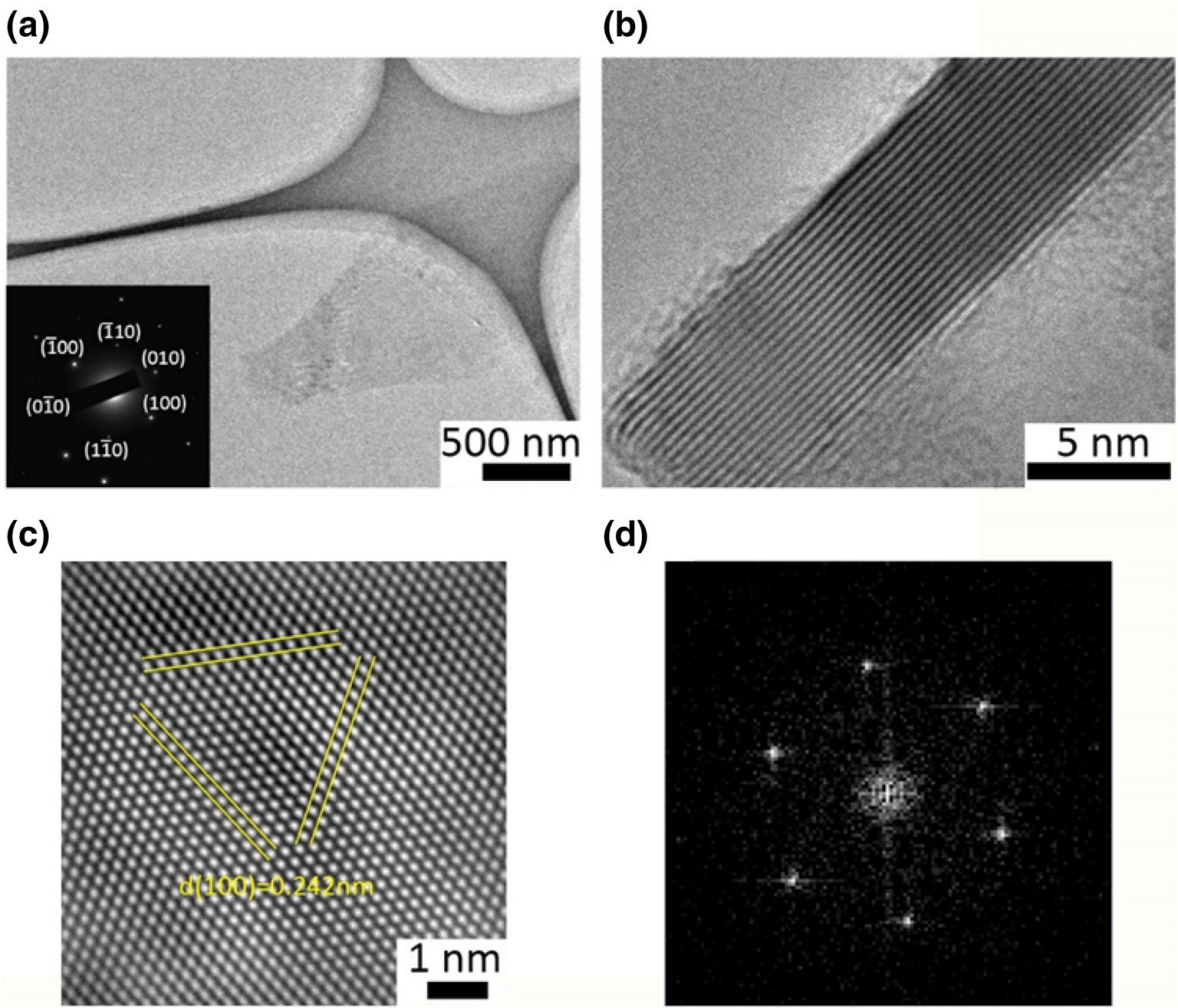
(**a**) Low-magnification bright-field TEM image of the FG specimen. The inset is the corresponding selected-area electron diffraction pattern showing the primarily single crystalline nature. (**b**) High resolution TEM image of the edge of the flakes consisting approximately 16 layers. (**c**) Fourier filtered high resolution TEM image of the FG and (**d**) the corresponding FFT image.

Figures 4 and 5 show the TEM images of PG and FG, respectively. The size of the sheets are in the 0.5–4 µm range. Insets in Figs. 4a and 5a are the corresponding [0001] SAED patterns. This shows that distinct six-fold symmetry diffraction spots are present more frequently in the FG specimen when compared to those of the PG sample. Moreover the electron diffraction pattern of the PG exhibit rather ring-like reflections (polycrystalline), as shown in the inset of Fig. 5a. It indicates that the FG shows either high crystallinity or/and without rotational boundaries in the observed area. The lattice parameters of PG and FG are 0.247 nm and 0.246 nm, respectively. A commonly used method to quantify the number of layers in graphite/graphene is by counting the number of folds at the edge of the flakes/sheets in high-resolution TEM images (HRTEM) as shown in Figs. 4b and 5b. This counting gives more than 68 layers in PG whereas FG has only 16 layers. The calculated interlayer distance of PG sheet is 0.339 nm, which is corresponding to (002) graphite crystal spacing. However, FG has a relatively smaller interlayer spacing of 0.326 nm. This is consistent with the XRD results in which FG’s (002) peak shifted toward the higher 2θ angle resulting in a smaller lattice spacing.

To distinguish the features more efficiently we remove the contrast due to the presence of amorphous materials, in Figs. 4c and 5c by the Fast Fourier Transform (FFT) method. The corresponding FFT images are shown in Figs. 4d and 5d for PG and FG respectively. It should be noted that the HRTEM images were taken at the featureless regions which may contain a few-layer graphene. The interplanar spacing along (100) planes obtained are 0.230 nm and 0.242 nm for the PG and FG, respectively, which are closely matching the lattice parameters calculated from the diffraction patterns. However, it is important to note that the periodicity in the region might not be the original position of the spots due to the instrumental limitations.

**Figure 6:**
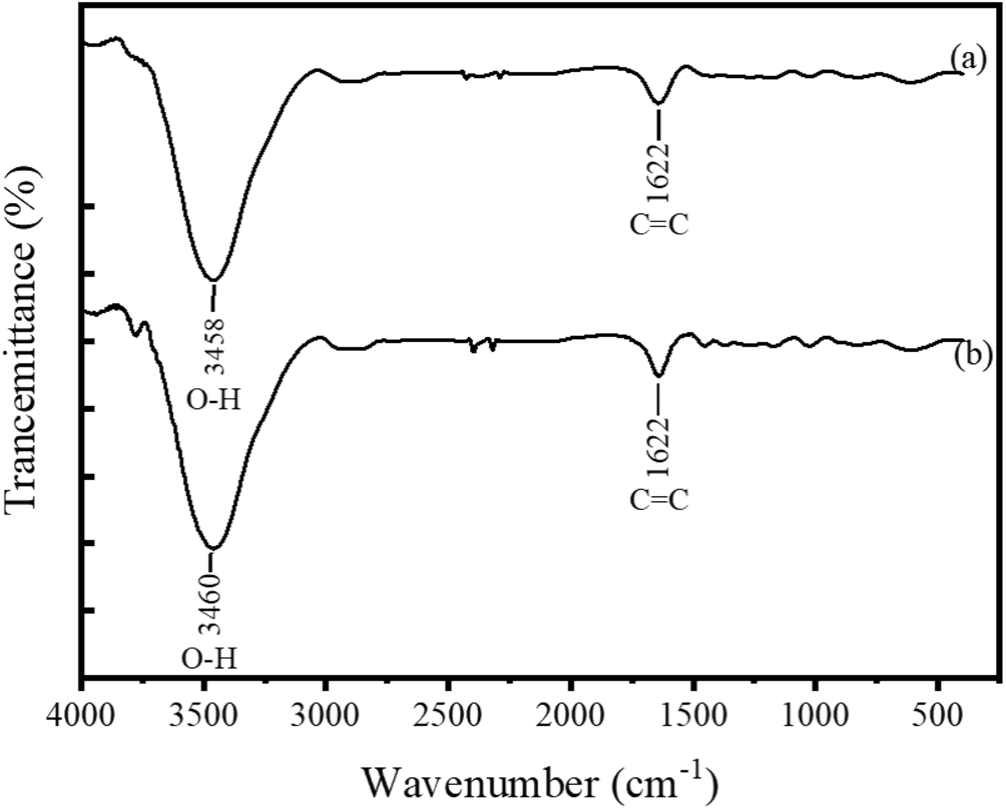
FT-IR spectra of (**a**) powdered graphite (PG) and (**b**) floated graphite (FG).

Figure 6 depicts the FT-IR spectra of PG and FG samples. The spectra of both samples are basically very similar with broad band between 3200 to 3700 cm^−1^ resembling the O–H vibrations of adsorbed water molecules on graphite surfaces and narrow band centered at 1622 cm^−1^ due to C=C stretching of conjugated double bonds that are present in graphene layers. The absence of bands at 2925 cm^−1^ (asymmetric C–H stretching in CH_2_ groups) 2855 cm^−1^ (symmetric C–H stretching in CH_2_ groups) suggests that there are no detectable amounts of saturated sp^3^ carbon atoms in both PG and FG samples. In other words, there are no noticeable defects due to hydrogenated double bonds in both samples. The absence of C=O vibration centred at 1738 cm^−1^ shows that the unsaturated carbon atoms do not contain any carbonyl functionality and hence the materials contain only conjugated C=C in their graphene sheets.

**Figure 7:**
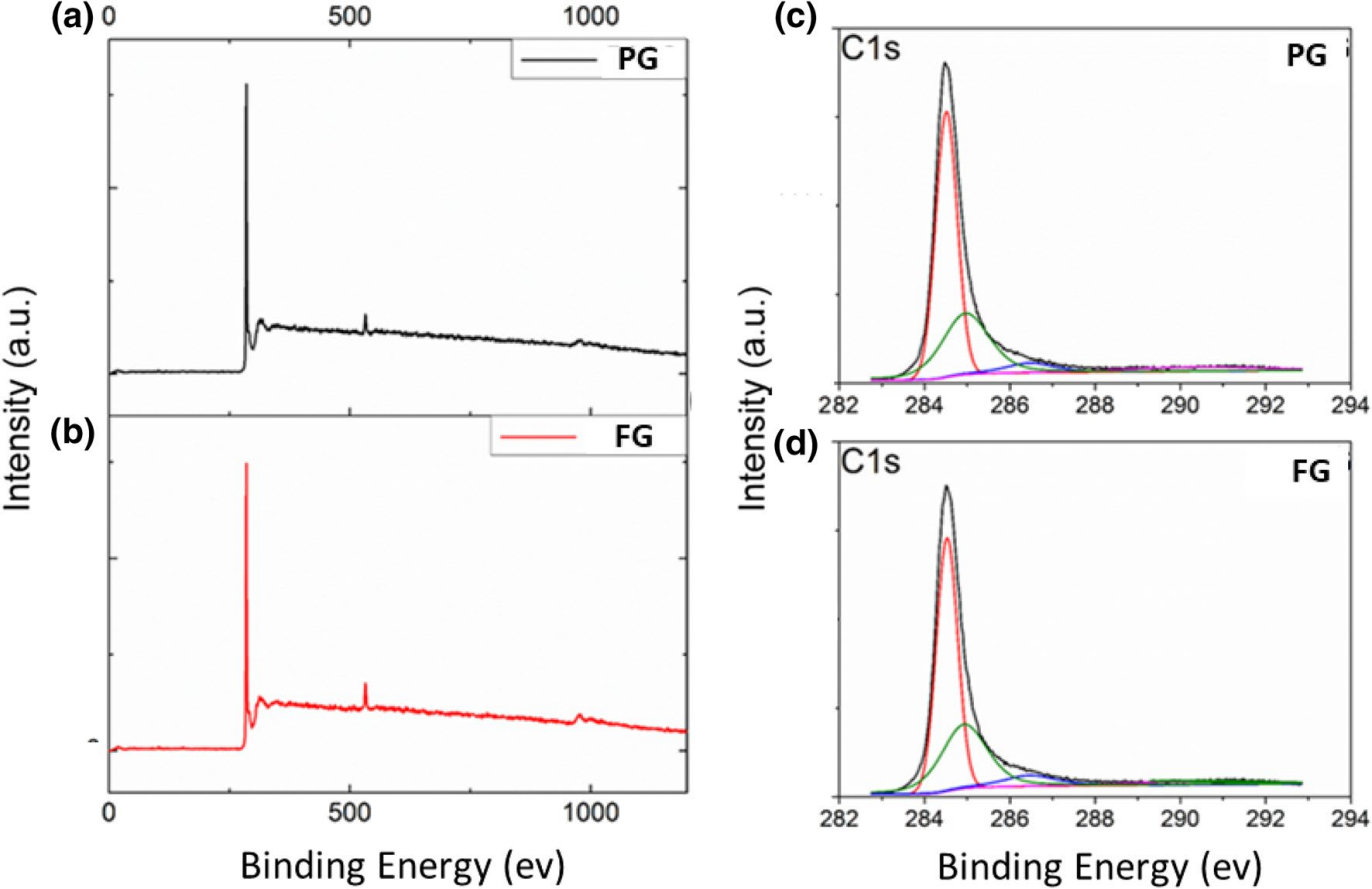
XPS survey spectra of (**a**) PG and (**b**) FG samples. Deconvoluted C1s spectra of (**c**) PG and (**d**) FG samples.

XPS was used to monitor the chemical state of carbon in both PG and FG samples. The survey scan spectra (Fig. 7) of PG (a) and FG (b) samples indicate the presence of C and O as major elements. The oxygen to carbon ratios (O/C) of PG and FG samples are 0.04 and 0.05, respectively. The C ^1^s spectra of the PG and the FG samples are shown in Fig. 7c,d. Both are fitted with four Lorentzian-Gaussian peaks of 20:80 ratios^24^. The most intense peak, at 284.5 eV, is assigned to sp^2^ C=C bonds together with the weak component at 290.1 eV that corresponds to its π–π transition (signature of graphitic carbon). The peak at 285 eV is due to sp^3^ C–C bonds. The component at 286.5 eV can be attributed to C–O–C/C–O bonds (ether and hydroxyl bonds, respectively). It is noted that the two samples exhibit similar patterns. However, FG has slightly higher percentage of the C–O and C–C components and lower percentage of the C=C and π–π bonds than PG. With respect to PG there is 4% reduction of C=C bonds, 3% increase in C–C and 11% increase in C–O–C in FG. It is generally accepted that C–O bonds can be formed at the edges of the graphene sheets. These edge functionalities can also be a reason for the increased intensity of the D peak in the Raman spectrum of FG.

The present study is the first attempt to understand the characteristics of the floated graphite in de-ionized water. This simple floatation technique has been introduced to float ground graphite particles with mineral impurities on the surface of de-ionized water, and floatation has been affected with the aid of shaking. This technique does not require any froth formers or floatation aiding chemicals and as such it is highly industrially viable and environmentally friendly. Furthermore, the complex purifying methods used in the literature and the graphite industry are tedious involving large number of processes which consume more energy^25–27^. Because of the low chemical and labour costs involved in the process described here, manufacturing of pure graphite is substantially less expensive and the innovative laboratory approach can be simply transferable to the industrial scale without having to alter to suit to industrial requirements.

The powdered and floated graphite samples have some differences in their crystallite sizes, morphologies, and purities. Floatation has resulted in the shrinking of crystallite size due to removing mineral inclusions within the interlayer spaces. The number of defects has been increased in floated graphite and both samples have some C–O–C ether bonds but in slightly higher amounts in the FG. The number of layers present in crystallites has been remarkably decreased in floated graphite compared to that of PG. The floatation technique can remove some impurities which are present as mineral inclusions in graphite. The floatation aids the remarkable enhancement of carbon content in graphite samples. It is more prominent when the original samples are impure with lower carbon percentages, leading to 4–12 times high-value addition to graphite. This technique can be applied to vein graphite rich in silicate minerals (especially along the wall zones of graphite veins) which are considered a waste of mining products. Further purification of the final graphite product is possible by removing iron components of the final FG product by magnetic separation and using very fine graphite powders for the flotation process.

## Methods

### Materials

Vein graphite samples collected from Bogala mines, Sri Lanka, were selected for this study. Morphologically different samples such as (a) flakes of radial graphite (b) flakes of striated graphite and (c) needle-platy graphite attached to the wall rock were subjected to the study.

### Purification of graphite by floatation method

**Figure 8:**
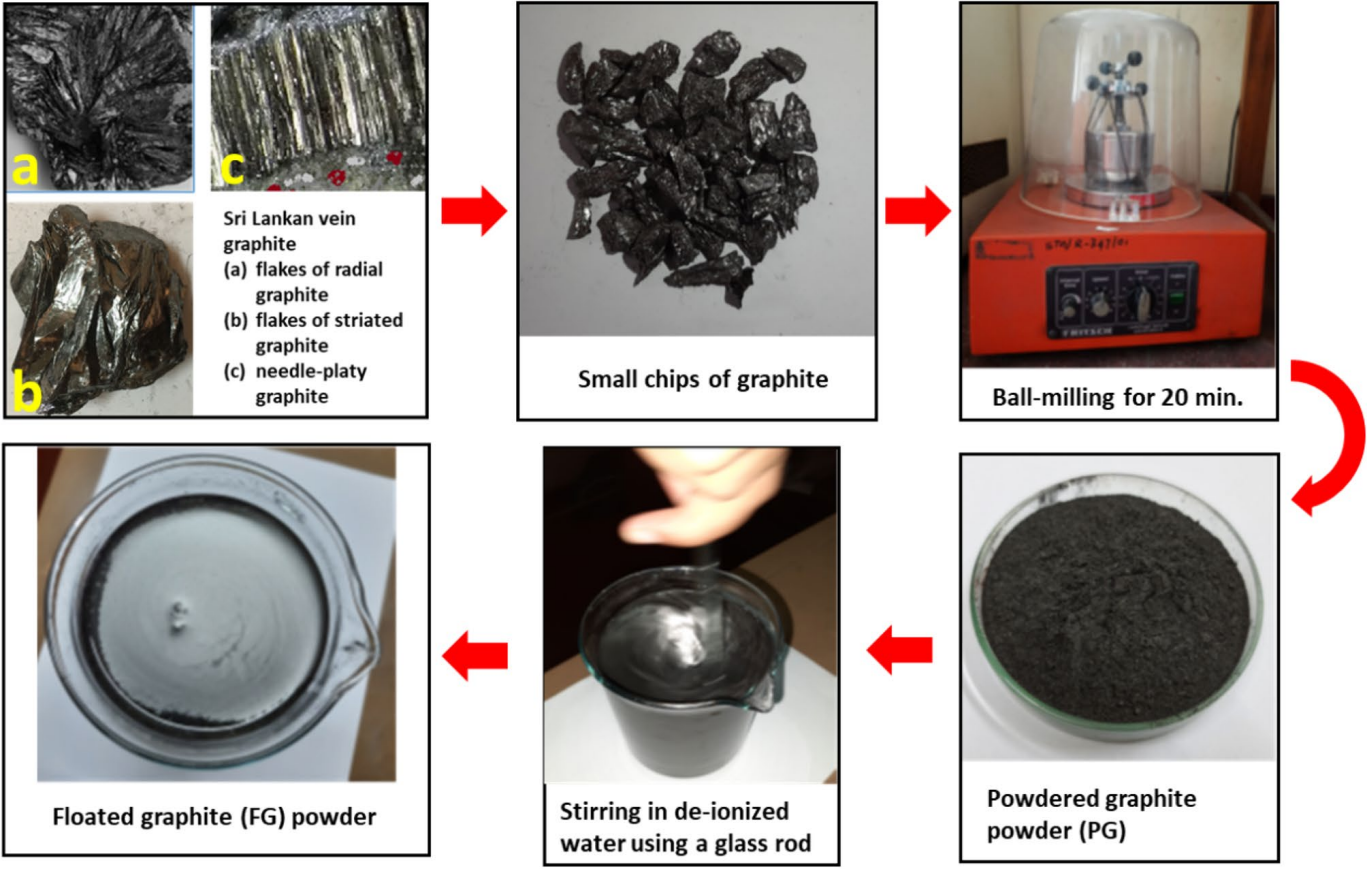
The process flow diagram of obtaining floated graphite from Sri Lankan vein graphite.

Figure 8 shows the process flow diagram using photographs taken at each step. Coarse samples from each variety were broken into small chips and crushed in a ball-mill for 20 min to form powdered graphite (PG). The grinding time was deliberately selected to be short to prevent the changes that may result in graphite structure due to mechanical shear. A portion of 5.0 g of particle size fraction less than 63 μm added to 500 mL of deionized water, at room temperature while stirring continuously. The fine graphite particles are then dispersed in the solution and coarser particles settled at the bottom of the container. A glass rod was dipped in the suspension and the suspension was shaken by rotating the glass rod about the vertical axis. Then the fine particles move upwards with the air bubbles generated. These fine particles were eventually floated on the surface of the water. This flotation technique does not require any chemicals for froth formation nor does it require chemicals for specific gravity adjustments.

### Characterization

X-ray diffraction (XRD) patterns were recorded using a powder diffraction machine, Rigaku, (RINT-TTR III), under Cu Kα radiation (λ = 1.5406 Å). Raman spectroscopic measurements were taken using a Renishaw InVia Reflex Raman microscopy system with a 532 nm laser. Fourier transform infrared (FT-IR) spectra were collected using an IR Prestige-21 Shimatzu FT-IR spectrophotometer using the KBr pellet method. Here, each sample was ground-well and homogenized. The samples were mixed with dry KBr in 1:40 mass-ratio to make the pellets. The transmittance of FT-IR spectra of the sample pellets were recorded by using a pure KBr pellet as the blank. Field-emission scanning electron microscopy along with energy-dispersive X-ray spectroscopy (EDS) was done by FE-SEM, Hitachi S-4700. Transmission electron microscopic (TEM) images were obtained on a JEM-2100 electron microscope (JEOL Ltd. Japan), at an accelerating voltage of 200 kV. The specimen was prepared by dispersing graphite powders in ethanol to form a suspension followed by ultrasonication for 1 h. A drop from each sample was put, separately, on carbon film-coated copper grids for observation. The high-resolution images of periodic structures were analysed and filtered by the Fast Fourier Transformation (FFT) method. X-ray photoelectron spectroscopic (XPS) measurements were performed using PHI 5000 VersaPribe II ESCA with a monochromated Al Kα radiation (1486.6 eV) available at the Toyota Technological Institute, Japan. Full scans (Binding energies ranging from 0 to 1200 V) were acquired using 1 eV/step, while the higher resolution scans were obtained using 0.025 eV/step. The pressure during the data acquisition was less than 1 × 10^–8^ Torr. The experimental curves were fitted using the Multipack data analysis software. The chemical composition of graphite samples was analysed by the Inductively Coupled Plasma Mass Spectrophotometry (ICP-MS- Perkin Elmer Sciex ELAN- 6000) at Activation Laboratories Ltd., Ontario, Canada. Detection limits for the ICP-MS were ranged between 0.2–0.001 ppm for trace elements.
